# Westminster Hospital

**Published:** 1852-11

**Authors:** 


WESTMINSTER HOSPITAL.—*Consulting Physician*—Dr. Bright. *Physicians*—Drs. Roe, Kingston, and Basham. *Assistant Physician*—Dr. Woodfall. *Consulting Surgeons*—Messrs. Guthrie and Hale Thomson. *Surgeons*—Messrs. Lynn, Phillips, and Holt. *Assistant Surgeon*—Mr. C. G. Guthrie. *Surgeon Dentist*—Mr. Clendon.

MEDICAL AND SURGICAL SCHOOL. *Winter Session.*—Anatomy and Physiology, Messrs. Hillman and Brooke. Anatomy, Descriptive and Surgical, Mr. Holthouse. Anatomical Demonstrations, Mr. Power. Surgery, Messrs. Phillips and Holt. Medicine, Drs. H. Roe and Basham. Chemistry, Mr. Lewis. Dental Surgery, Mr. Clendon. *Summer Session.*—Materia Medica, Dr. Basham. Midwifery, Dr. F. Bird. Forensic Medicine, Drs. Fincham and Tanner. Botany, Dr. Radcliffe. Practical Chemistry, Mr. H. Lewis. Natural Philosophy, Mr. H. Brooke.

